# Integrating expert opinions with clinical trial data to analyse low-powered subgroup analyses: a Bayesian analysis of the VeRDiCT trial

**DOI:** 10.1186/s12874-020-01178-6

**Published:** 2020-12-10

**Authors:** Russell Thirard, Raimondo Ascione, Jane M. Blazeby, Chris A. Rogers

**Affiliations:** 1 Bristol Trials Centre (BTC), University of Bristol, Zone A, level 7, Bristol Royal Infirmary, Bristol, BS2 8HW UK; 2 Bristol Heart Institute, Bristol Medical School, University of Bristol, Bristol, UK; 3 National Institute for Health Research Bristol Biomedical Research Centre, Bristol, UK; 4 Division of Surgery, University Hospitals Bristol NHS Foundation Trust, Bristol, UK

**Keywords:** Bayesian analysis, Subgroup analyses, Survival, Cardiac surgery, Volume replacement therapy, Elicitation

## Abstract

**Background:**

Typically, subgroup analyses in clinical trials are conducted by comparing the intervention effect in each subgroup by means of an interaction test. However, trials are rarely, if ever, adequately powered for interaction tests, so clinically important interactions may go undetected. We discuss the application of Bayesian methods by using expert opinions alongside the trial data. We applied this methodology to the VeRDiCT trial investigating the effect of preoperative volume replacement therapy (VRT) versus no VRT (usual care) in diabetic patients undergoing cardiac surgery. Two subgroup effects were of clinical interest, a) preoperative renal failure and b) preoperative type of antidiabetic medication.

**Methods:**

Clinical experts were identified within the VeRDiCT trial centre in the UK. A questionnaire was designed to elicit opinions on the impact of VRT on the primary outcome of time from surgery until medically fit for hospital discharge, in the different subgroups. Prior beliefs of the subgroup effect of VRT were elicited face-to-face using two unconditional and one conditional questions per subgroup analysis. The robustness of results to the ‘community of priors’ was assessed. The community of priors was built using the expert priors for the mean average treatment effect, the interaction effect or both in a Bayesian Cox proportional hazards model implemented in the STAN software in R.

**Results:**

Expert opinions were obtained from 7 clinicians (6 cardiac surgeons and 1 cardiac anaesthetist). Participating experts believed VRT could reduce the length of recovery compared to usual care and the greatest benefit was expected in the subgroups with the more severe comorbidity. The Bayesian posterior estimates were more precise compared to the frequentist maximum likelihood estimate and were shifted toward the overall mean treatment effect.

**Conclusions:**

In the VeRDiCT trial, the Bayesian analysis did not provide evidence of a difference in treatment effect across subgroups. However, this approach increased the precision of the estimated subgroup effects and produced more stable treatment effect point estimates than the frequentist approach. Trial methodologists are encouraged to prospectively consider Bayesian subgroup analyses when low-powered interaction tests are planned.

**Trial registration:**

ISRCTN, ISRCTN02159606. Registered 29th October 2008.

**Supplementary Information:**

The online version contains supplementary material available at 10.1186/s12874-020-01178-6.

## Background

In clinical trials, evaluation of intervention effects in subgroups of patients may be pre-specified in the study protocol, or exploratory analyses carried out post-hoc. The aim of such analyses is to assess if the intervention effect is consistent across patients or if specific subgroups of patients experience larger benefit or harm. Typically, subgroup analyses in clinical trials are conducted by comparing the intervention effect in each subgroup by means of an interaction test in the frequentist framework. In trials reporting subgroup analyses, between 27 and 34.5% planned a statistical test for interaction [1–3]. When the test does not provide evidence of an interaction, it is recommended to report the overall results. However, these tests are low-powered and clinically important interactions in subgroup of patients may go undetected. In light of this, some suggest raising the Type I error rate when testing interactions, thereby increasing power but this is offset by the increased risk of “false positives” and chance findings [4].

A Bayesian analysis differs from the frequentist analysis in that the uncertainty about unknown parameters such as an interaction parameter can be expressed in a ‘prior’ distribution. Instead of assuming that there is no prior knowledge about the interaction effect, or assuming that there is no interaction by pooling all patients together irrespective of their subgroup, the proposed Bayesian approach allows the user to flexibly apply an expert informative prior for the interaction parameter using external knowledge to the trial [5]. Data from previous studies and/or expert opinions can be used in a prior to characterise the possibly differential effects of an intervention for subgroups of patients having different comorbidities. By Bayes’ theorem, inferences about the subgroup treatment effects are drawn from the ‘posterior’ distributions. The posterior distributions are proportional to the product of the expert prior distributions and the trial data also referred as the ‘likelihood’. The interest of this method lies in situations where increasing the power by collecting more patient-level data is not feasible, too expensive or too time-consuming.

### Motivating case study

The VeRDiCT study comprised two randomised controlled trials conducted in parallel. The study investigated the effect of volume replacement therapy (VRT) on postoperative length of stay before being fit-for-discharge. The population of interest were diabetic patients undergoing coronary artery bypass grafting surgery. The study randomised 169 patients (122 in the UK and 47 in India). Two subgroup effects were of clinical interest, a) preoperative renal function and b) preoperative blood glucose management. In relation to subgroup a), VRT effect was compared in patients with evidence of either microalbuminuria or diabetic nephropathy to patients without (low risk of renal failure subgroup A1 versus high risk subgroup A2). With respect to subgroup b), the analysis compared patients managing their diabetes only with oral medication (less severe comorbidity subgroup B1) to patients taking only insulin or combined with oral medication (more severe comorbidity subgroup B2). One hundred and seventy participants were required in order to detect a significant intervention effect in the combined trial population using frequentist methods. Recruiting as many in each subgroup was not feasible as the intervention is very specialised and would have extended the recruitment period by more than 4 years of recruitment. Hence, the aim of this study was to apply a Bayesian approach to analyse the subgroup effects in the VeRDiCT trial, and demonstrate how this approach can provide more power to detect treatment effects and additional insights to interpreting subgroup effects compared to the frequentist framework.

## Methods

### Prior elicitation and expert opinion derivation

An extensive literature is available on elicitation methods and associated heuristics that may bias an expert’s ability to assess probabilities [6–9]. Examples of elicitation questionnaires in medical trials have been published [10–13]. Our questionnaire was specifically produced for this study (see Additional file 1) and is based on the work of Spiegelhalter et al. [14]. Experts were identified as health professionals well informed about VRT and effects in diabetic patients undergoing CABG surgery. Cardiac surgeons and cardiac anaesthetists were identified within the UK trial site. The questionnaire was delivered face-to-face and as part of a routine research meeting before the results of the VeRDiCT trial were available. As hazards are complex to elicit, experts were asked about the number of patients they would expect to be fit-for-discharge within 6 days post-operation. Six days post-operation was determined as the median length of stay for diabetic patients undergoing CABG surgery between 2012 and 2015 from the Patient Analysis and Tracking System (PATS) database – a registry of every adult cardiac surgical procedure undertaken at the Bristol Royal Infirmary cardiac surgical unit. Being fit-for-discharge within 6 days was defined as the outcome in the questionnaire as a ‘normal’ recovery without any major complications.

The experts were asked to express their opinions in the quantile format as suggested by Cooke [15, 16]. Experts provided percentiles of their subjective distribution using the median, 2.5 and 97.5 percentiles (i.e. the most likely value, the lowest and highest plausible values respectively). Experts were encouraged to imagine as if they would have to provide bounds of a 95% range of plausible values.

The questionnaire answers were used as prior beliefs for the hazard ratios. Hence a transformation was required to convert the opinions to the log hazard ratio scale:

$$ \log (HR)=\log \left[\frac{\ \log\ \left(1-{p}_1\right)}{\ \log\ \left(1-{p}_2\right)}\right] $$

where *p*_1_ and *p*_2_ are the probabilities of having a normal recovery in the VRT group and usual care group respectively.

A positive log(*HR*) (i.e. HR > 1) suggests that the ‘hazard’ of having a normal recovery is higher and therefore, favours the VRT group compared to the usual care group. The log(*HR*) was assumed to follow an approximate normal likelihood distribution. Each expert’s elicited opinions were fitted with normal distributions using least squares on the cumulative distribution function [17]. The experts’ individual distributions were then aggregated to obtain a combined experts prior distribution using the linear opinion pooling method [18].

The intervention effects on the log hazard ratio scale are denoted *θ*_1_ and *θ*_2_ for the less and more severe subgroups respectively such that ${\theta}_1\sim N\left({p}_1,{s}_1^2\right)$ and ${\theta}_2\sim N\left({p}_2,{s}_2^2\right)$. Experts were firstly asked to provide their opinions about the number of patients treated with VRT having a normal recovery in the less and more severe subgroups separately in each unconditional question. This was then followed by a conditional question which asked the experts how their opinions changed about the effect in the more severe subgroups given two scenarios in the less severe subgroups. The two scenarios were: a) if we knew the true effect in the less severe subgroup is null (i.e. *θ*_1_ = 0); and b) if we knew the true effect in the less severe subgroup is beneficial by a value *d* (i.e. *θ*_1_ = *d*, *d* > 0). The following distributions were derived: ${\theta}_2\mid \left({\theta}_1=0\right)\sim N\left({p}_{20},{s}_{20}^2\right)$ and ${\theta}_2\mid \left({\theta}_1=d\right)\sim N\left({p}_{2d},{s}_{2d}^2\right)$. For the risk of renal failure subgroup analysis, the beneficial change was $d=\log \left[\frac{\log \left(1-70/100\right)}{\log \left(1-60/100\right)}\right]$ meaning we supposed that in truth VRT increased the number of patients with low risk of renal failure having a normal recovery to 70 compared to 60 when treated with usual care. For the blood glucose management subgroup analysis, the change was $d=\log \left[\frac{\log \left(1-65/100\right)}{\log \left(1-50/100\right)}\right]$. The joint prior distribution of the treatment effect in each subgroup $$ \left(\begin{array}{c}{\theta}_1\\ {}{\theta}_2\end{array}\right) $$ was modelled as a bivariate Normal with mean *m* = (*p*_1_, *p*_2_) and variance-covariance matrix $$ V=\left(\begin{array}{cc}{V}_{11}& {V}_{12}\\ {}{V}_{12}& {V}_{22}\end{array}\right) $$. The parameter derivations followed the methods outlined by White et al. [19]:

1. The unconditional variance *V*_11_ for the effect of VRT in the less severe subgroup was derived from the variance of the elicited distribution: ${V}_{11}={s}_1^2$.
2. The covariance element *V*_12_ was derived as *V*_12_ = *b*_12_*V*_11_. The regression coefficient (*b*_12_) of the treatment effect in the more severe subgroup on the treatment effect in the less severe subgroup is defined as ${b}_{12}=\frac{p_{2d}-{p}_{20}}{d-0}$.
3. The variance of the treatment effect in the more severe subgroup *V*_22_ is derived such that ${V}_{22}=\mathit{\operatorname{var}}\left({\theta}_2|{\theta}_1\right)+{b}_{12}^2\ {V}_{11}$. The variance of the treatment effect in the more severe subgroup conditional on the treatment effect in the less severe subgroup was derived as the average of the two conditional question variances: $\mathit{\operatorname{var}}\left({\theta}_2|{\theta}_1\right)=\frac{s_{20}^2+{s}_{2d}^2}{2}$. A sensitivity analysis assuming the variance ${V}_{22}={s}_2^2$ was performed to compare posterior estimates to alternative variance derivations when the resulting variance-covariance was positive definite.
4. A further sensitivity analysis was performed by drawing the mean of *θ*_2_ from the regression *E*(*θ*_2_| *θ*_1_) = *p*_20_ + *b*_12_*θ*_1_, giving *E*(*θ*_2_) = *p*_20_ + *b*_12_*p*_1_.

### Community of prior specifications

This paper assessed results from a wide variety of expert opinions and ways of using the expert opinions. A clinical prior, a sceptical prior, an interaction prior, and other specifications of the expert opinions composed our ‘community of priors’ and are described below:

- The clinical prior was directly derived from the experts’ opinions: *θ*~*N*_2_(*m*, *V*)
- The sceptical prior used the experts’ opinions for the variance component, but the mean was centred around the null effect: *θ*~*N*_2_(0, *V*)
- The interaction prior only used an informative expert prior for the treatment-by-subgroup interactions. This was enabled by re-parameterising *θ* such that the prior *ψ* consisted of an uninformative prior for the mean average VRT effect and an informative prior for the interaction term comparing the effect in the more severe subgroup to that in the less severe subgroup:
  $$ \psi =C\ \theta =\left(\begin{array}{cc}1/2& 1/2\\ {}-1& 1\end{array}\right)\times \left(\begin{array}{c}{\theta}_1\\ {}{\theta}_2\end{array}\right)=\left(\ \begin{array}{c}\frac{1}{2}\left({\theta}_1+{\theta}_2\right)\\ {}{\theta}_2-{\theta}_1\end{array}\right) $$
  The prior *ψ* variance is *W* = *CVC*^*T*^. The matrix *W* was modified such that: $$ {W}^{\ast }=\left(\begin{array}{cc}L& 0\\ {}0& {W}_{22}\end{array}\right) $$ with *L* an arbitrarily large number. *L* in W^*^ expresses the lack of information about the average treatment effect whilst *W*_22_ expresses the prior information from our experts about the interaction effect *θ*_2_ − *θ*_1_. The off-diagonal elements express the absence of any covariance beliefs between the average intervention effect and the interaction effect. A matrix *V*^∗^ was back transformed such that *V*^∗^ = *C*^−1^W^*^C^−*T*^. The interaction prior followed *θ*~*N*_2_(*m*, *V*^∗^). The Bayesian analysis using the interaction prior specification was defined as the primary analysis of the VeRDiCT trial.
- A further specification of the interaction prior *θ*~*N*_2_(0, *V*^∗^) was assessed to avoid reporting qualitative interactions (e.g. *θ*_1_ < 0 and *θ*_2_ > 0) in specific cases where the treatment effects were null in both subgroups but the experts believed VRT was much better in the more severe subgroup than in the less severe subgroup (i.e. *θ*_2_ − *θ*_1_ > 0).
- A vague prior that would closely reproduce the results of a frequentist maximum likelihood estimate (MLE): *θ*~*N*_2_(0, *LI*) where *L* is an arbitrarily large number and *I* is the identity matrix. This prior specification was added following comments from clinical experts asking about the performance of the Bayesian approach when there is no knowledge about the subgroup effects. Table 1 presents a summary of the community of priors and their associated mean and variance parameters used to assess posteriors results.

**Table 1:** Summary of prior specifications

Prior specifications	Mean parameter	Variance parameters	
		Mean effect	Interaction effect
Clinical	m	V^a^	V^a^
Sceptical	0	V^a^	V^a^
Interaction	m	V*^b^	V*^a^
Interaction-variance	0	V*^b^	V*^a^
Vague	0	LI^b^	LI^b^

^a^Informative using expert opinions ^b^Uninformative

### Statistical analysis

The VeRDiCT primary outcome was time-to-event and was analysed using an adjusted Cox proportional hazards model in the trial report [20]. A Bayesian counterpart of the Cox proportional hazard model was performed adjusting for the same covariates. Analyses were performed using STAN software from R [21, 22] and STATA version 15.1 (StataCorp LP, College Station, TX, USA). White et al. proposed a normal-normal conjugate Bayesian analysis as the number of events was large and therefore the log-likelihood was reasonably approximated by a normal distribution [19]. However, we did not take this approach but jointly estimated the log-likelihood and the prior in a fully Bayesian Cox model to extend the proposed method to scenarios of low number of events. The STAN code for this analysis is provided in the supplementary materials (see Additional file 2). As the experts were all from the UK site, the analysis was restricted to the UK trial. The results of the Bayesian analysis were compared with the frequentist approach to assess relative merits of each statistical framework.

## Results

### VeRDiCT trial and expert results

**Fig. 1:**
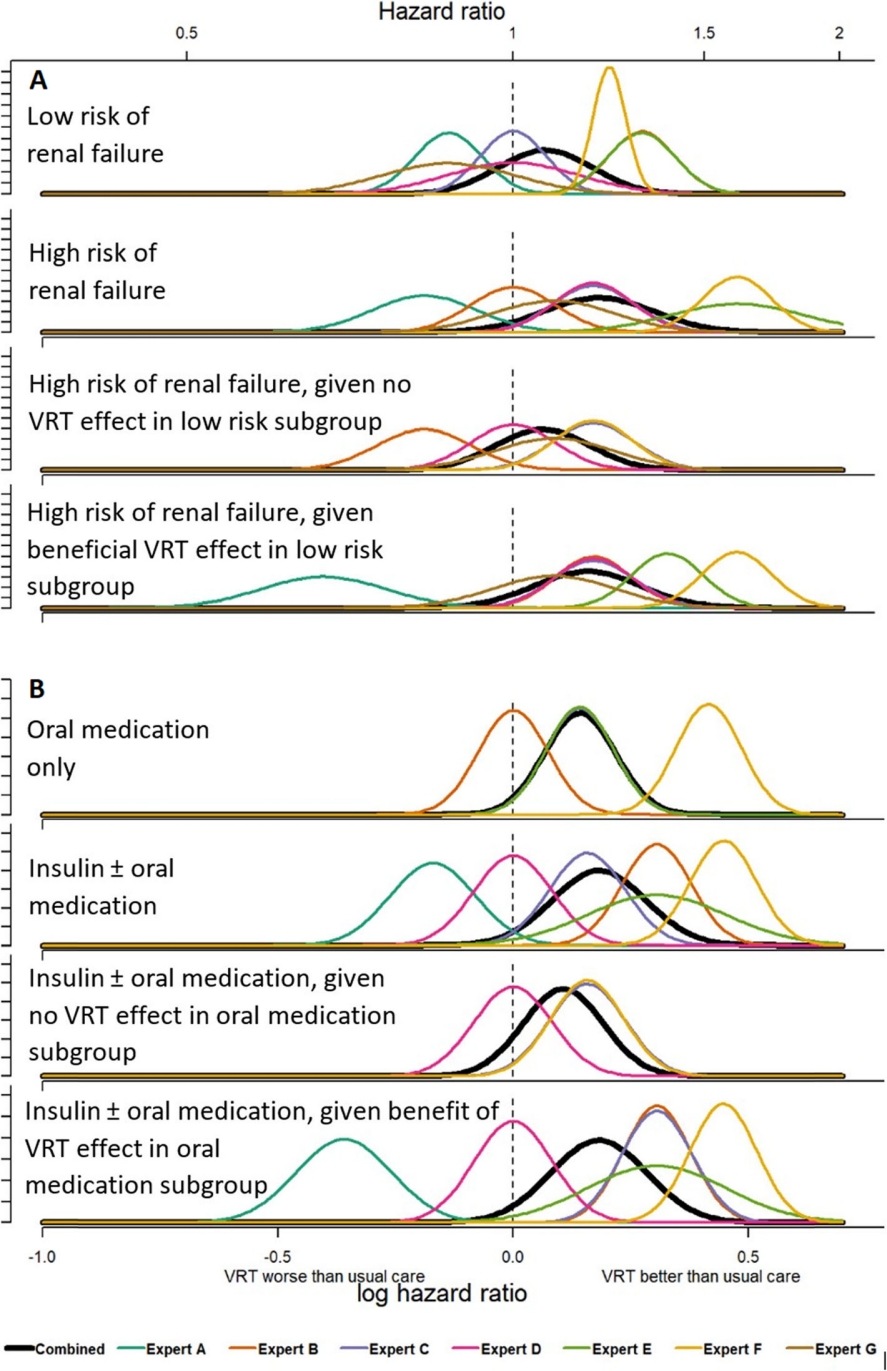
Elicited prior distributions of the log hazard ratio. The elicited prior distribution are presented for each expert and for the combined prior, for the risk of renal failure subgroups (**a**) and for the type of antidiabetic medication subgroups (**b**)

Table 2 presents baseline characteristics of the 121 participants who underwent randomisation in the UK trial of the VeRDICT study. The baseline characteristics were similar across treatment groups. The pre-surgery blood glucose management presents a slight imbalance with a higher proportion of patients managing their blood glucose with oral medication in this those randomised to VRT compared to patients randomised to usual care. Apart from the opinion of expert A, the unanimous belief was that VRT was better or at least not worse than usual care for all subgroups of patients (Fig. 1). Additionally, the experts believed that VRT would provide the greatest benefit for higher risk patients with more severe comorbidities. For example, patients with high risk of renal failure or patients treated with insulin ± oral medication were expected to have 20% higher ‘hazard’ of a normal recovery with VRT compared to usual care (HR = 1.20, 95% CI 0.95–1.51 and HR = 1.20, 95% CI 0.99–1.46 respectively). Nonetheless, the combined experts’ prior (presented in black) still includes the null effect suggesting there is some uncertainty about VRT being better than usual care

**Table 2:** Baseline characteristics of the UK VeRDiCT trial participants

Treatment allocation	Randomised to usual care (*n* = 61) n (%)	Randomised to VRT (*n* = 60) n (%)
*Minimisation criteria*		
Age > 70 years	17 (28)	17 (28)
Female gender	10 (16)	10 (17)
Preoperative creatinine > 160 μmol/L	3 (5)	4 (7)
Ejection fraction < 50%	49 (80)	49 (82)
Cardiac angiogram in the 5 days prior to surgery	5 (8)	7 (12)
*Subgroup variables*		
Low risk of renal failure	37 (62)^a^	38 (63)
Oral diabetic medication only	30 (49)	35 (58)

^a^One patient with missing data

### Bayesian analysis results

**Fig. 2:**
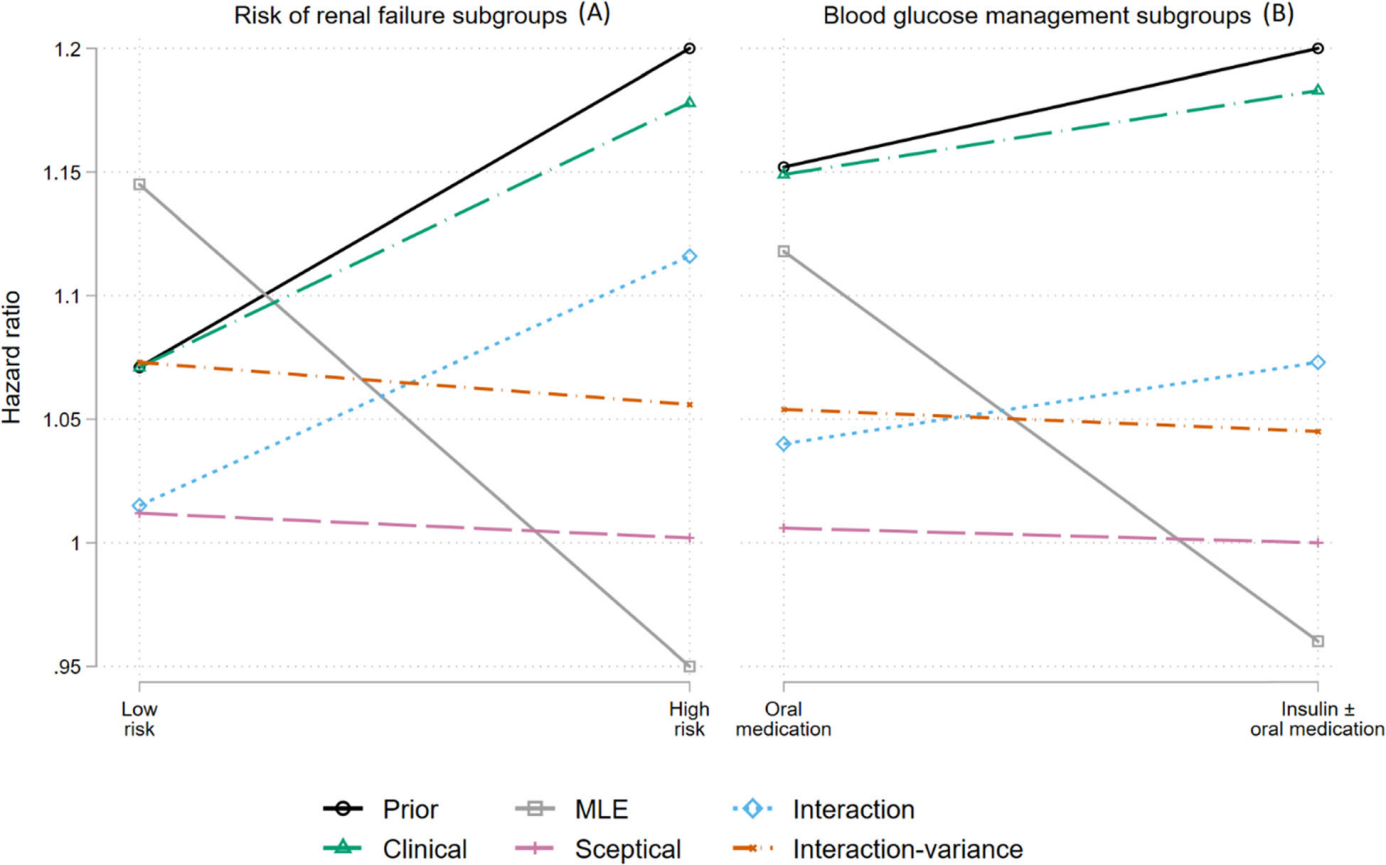
Expert prior median and posterior median estimates using the pooled expert prior with different specifications

The results of the Bayesian analyses combining the trial data and the expert opinions were very similar for both subgroup analyses A and B: the trial data treatment estimates (MLEs) and the expert opinions (prior medians) were of the comparable magnitude and direction in the risk of renal failure analysis (left panel) compared to the blood glucose management subgroup analysis (right panel) in Fig. 2. Hence for the following results, we have focused on the risk of renal failure subgroup analysis.

While the experts believed that VRT would be of greater benefit in the higher risk subgroup (shown in black), this was not borne out of the trial data (grey); the maximum likelihood hazard ratio was lower in the high-risk group than in the low-risk group. The posterior results using an expert prior for the interaction effect but a vague prior for the mean effect (i.e. the interaction prior shown in blue) produces a slope that is similar to that of the prior but shifted towards the trial’s mean treatment effect (HR = 1.07). The clinical posterior estimates are closely aligned to the prior, in location and in slope, as it uses the expert beliefs for both the overall mean and the interaction effects. This result suggests the clinical prior dominates the trial data when using informative priors for both the interaction effect and the mean effect. The slopes of the posterior estimates using the sceptical and the interaction-variance priors lie between the MLE and the prior, with their location centred around the null effect and the mean treatment effect respectively.

**Fig. 3:**
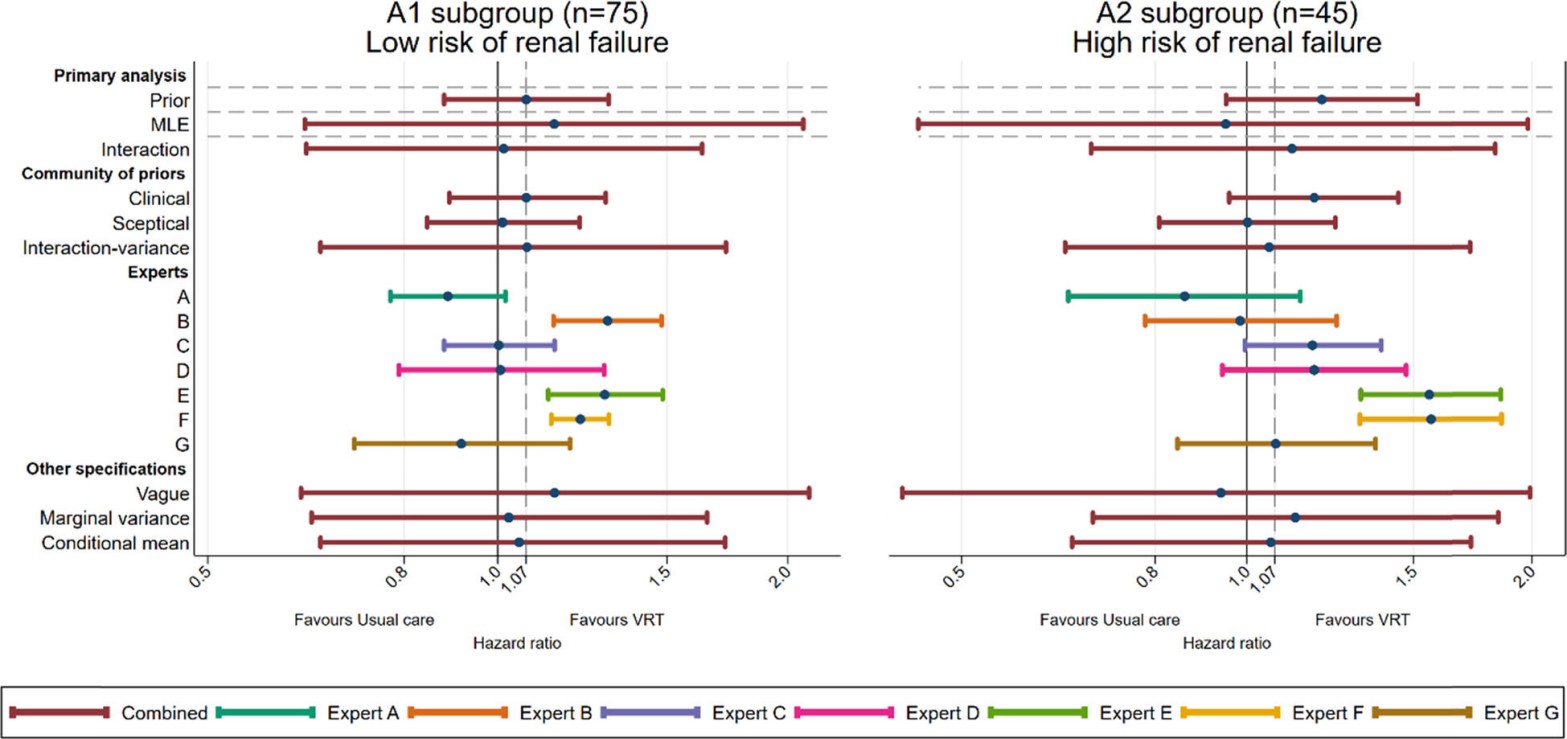
VeRDiCT Bayesian results: intervention effect comparing VRT to usual care in subgroups A. Prior median and 95% Interval, MLE estimate and 95% confidence intervals, and posterior medians and 95% credible intervals for the treatment effect in the risk of renal failure subgroups are presented

Figure 3 presents the prior median, MLE and posteriors estimates for the risk of renal failure subgroup analysis and their variability. In this trial, the 95% confidence intervals for MLE are wide (e.g. 95% CI 0.63–2.08 and 95% CI 0.45–1.98 for A1 and A2 subgroups respectively) due to small subsamples (75 patients in subgroup A1, 45 in A2, 65 in B1 and 56 in B2). The variability of the combined experts’ prior distribution was lower than the variability in the trial treatment effect MLEs.

The Bayesian posterior results using expert beliefs have increased precision compared to the MLE. In the presence of a qualitative interaction (i.e. the treatment effects in the subgroups are in opposite directions) which was observed in the trial data MLEs, the posterior estimates using the interaction prior are pulled towards the mean treatment effect. The precision of the estimates is increased but the results do not provide enough evidence of a benefit of VRT (52 and 67% probability of VRT being better than usual care for less and more severe subgroup respectively). Using each expert prior individually gave posterior estimates which varied across experts. The posterior estimates using expert A’s prior, who was the most sceptical about VRT, favours usual care, with probabilities of only 5 and 15% of benefit with VRT in the less and more severe subgroups respectively. In contrast, experts E and F were very enthusiastic about the effect of VRT and the posterior estimate reports a 100% probability of VRT being better for both subgroups.

The posterior estimates using the vague prior confirms that when using uninformative priors for all the parameters, the results are consistent with the MLEs that only use trial data for inferences. Although the posterior results were robust to the different variance derivation, the posterior results using the conditional mean derivation returned point estimates that were different compared to the unconditional mean derivation (HR = 1.05 vs HR = 1.01 in the low risk subgroup and HR = 1.06 vs HR = 1.12 in the high risk subgroup). We argue the prior mean of *θ*_2_ derived from the experts’ answers to the second unconditional question is a direct derivation requiring less manipulation than the conditional mean derivation and therefore the unconditional method should be preferred. The posteriors results estimates are provided in the supplementary files (see Additional file 3).

## Discussion

A Bayesian approach to subgroup analyses has been successfully applied to the UK VeRDiCT trial. Expert opinions were elicited without knowledge of the trial results and these opinions were combined with the trial data under a range of prior specifications. The questionnaire eliciting opinions was challenging to design as it needed to not be overburdensome but at the same time capture the information required (i.e. personal opinions on the effect of VRT in each subgroup and the interaction effect between the intervention and the subgroups). Face-to-face elicitation had the advantage that training and feedback could be provided to the experts iteratively. Other elicitation methods (e.g. postal questionnaires) were considered but we chose a face-to-face approach to allow us the opportunity to answer questions and clarify what was being asked where required. As previously reported [23–25], experts experienced challenges when characterising a range of plausible values and the elicitation range may influence how they express their opinion. The Bayesian methods offer a formal framework to quantify clinical opinions and use them when relevant historical data is not available. It also provides an opportunity to assess how using the expert opinions under different assumptions of the community of priors impacts the results. The sensitivity of results to alternative expert opinion derivations were also assessed and were helpful in identifying whether various mean and variance parameter derivation choices impact the posterior estimates in the context of this trial.

The primary Bayesian analysis results using the interaction prior suggests there is insufficient evidence in favour of VRT being better than usual care. In this sense, the Bayesian analysis is consistent with the frequentist analysis and suggests further research is needed to claim any subgroup effects. The elicitation results indicated that experts believed VRT was better than usual care, and that the effect would be greatest for patients with more severe comorbidities. These opinions were not supported by the trial data, which suggested VRT was worse than usual care for participants with more severe comorbidities. It is unclear whether this inconsistency is due to ‘inaccurate’ expert opinions or spurious findings from the frequentist analysis which is susceptible to outliers given the small sample size.

Trial reporting guidelines advocate against presenting subgroups estimates if the interaction test is not significant [26]. However, this recommendation also prevents us from understanding the effect of an intervention in subgroups of interest. Using an informative prior for the interaction parameters (i.e. the interaction and interaction-variance priors) and an uninformative prior for the overall mean treatment effect in the Bayesian analysis allows us to draw inferences for both subgroups. By ‘borrowing’ information from the treatment effect in the complementary subgroup, subgroup posterior estimates using the interaction priors were more stable (less extreme point estimates and higher precision) than frequentist subgroup estimates. Our proposed Bayesian subgroup model shifts point estimates and their associated credible intervals towards the overall mean effect, whereas classical frequentist approaches keep the point estimate fixed and adjust for multiple comparisons by making the confidence intervals wider. Analyses modelling the treatment effects in a joint model have been reported to be sensible approaches to multiplicity as multiple treatment inferences are directly incorporated in the model [27, 28]. To maximise the use of the trial data, we encourage methodologists to prospectively consider Bayesian subgroup analyses using expert opinions when several low-powered subgroup analyses are planned.

Our study had several strengths but also some limitations. First, we have identified experts within one trial centre, which may have introduced bias insofar that our experts probably had similar opinions that may not reflect all experts’ opinions on the effect of VRT. Also, we restricted the analysis to the UK expert opinions and only included data from the UK centre in this case study. The rationale for this was that it was impractical to elicit experts from the centre in India. Ideally, opinions would be elicited from experts from several sites of a multicentre trial, which can then be incorporated in a hierarchical Bayesian model. Furthermore, we could have elicited opinions from more experts, although there is no consensus on the number of expert opinions required. Previous studies have used as few as one expert [29] and as many as 37 experts [10]. The number of experts will typically depend on the number of available experts in your field. Fewer thoughtful opinions are more valuable than a higher number of opinions from less engaged experts [30].

Second, it is recognised that the quality of the interviews is important in determining participant responses. We used a simple questionnaire and piloted it, but it is possible that a more in-depth interviews undertaken by a non-statistician would have elicited different responses. Audio recording interviews that are then independently assessed for quality a second opinion of their content could be a useful addition [31].

Third, the approach to Bayesian subgroup analysis reported here is similar to that conducted by White et al. [19]. In that study the expert opinions had little impact on the posteriors as each of the three subgroups were adequately powered to detect subgroup treatment effects. The Bayesian approach is of greatest value and impact when the study is under-powered for subgroup analyses.

Fourth, introducing prior expert opinions in several different specifications generated more precise posterior estimates and a shift of the point estimates compared to the MLEs in the VeRDiCT trial. An increase in precision and shift of the estimate away from the null increases the power to detect a treatment effect, but at the cost of bias in the treatment estimate. An extension of the proposed methodology could investigate how clinical opinions influence the posterior results of a Bayesian subgroup analysis when the true subgroup treatment effects are known. A simulation study could investigate the impact of the magnitude of the interaction effect and sample sizes on statistical inferences by assessing the trade-off between bias and power to detect a treatment effect.

## Conclusions

This Bayesian subgroup approach proves its value in cases where fully powered subgroup analyses are not feasible, time-consuming or too expensive. With limited resources, experts can be elicited, and their opinions can help maximise the trial data. Adding experts’ opinions to the analyses could increase the precision of the treatment estimate as we have noted in our motivating case study which could in return increase power to detect an effect. Still, and as any subgroup analysis, researchers need to be cautious in appraising each posterior distribution results, acknowledge the limitations of the findings, and provide supporting or contradictory data from other studies when available.

## Supplementary Information

**Additional file 1.** Elicitation questionnaire for the VeRDiCT trial.

**Additional file 2.** STAN model code.

**Additional file 3.** Point estimate and interval results of the Bayesian analysis of the VeRDiCT trial for subgroups A and B.

## Data Availability

The STAN code to implement the developed approach is available in the supplementary material. The data that support the findings of this study are not publicly available but anonymised individual patient data will be made available upon reasonable request to the authors for secondary research, conditional on assurance from the researcher that the proposed use of the data is compliant with the MRC Policy on Data Preservation and Sharing regarding scientific quality, ethical requirements and value for money. A minimum requirement with respect to scientific quality will be a publicly available pre-specified protocol describing the purpose, methods and analysis of the secondary research. The datasets used during the current study are available from the corresponding author on reasonable request.

## Abbreviations

CABG: Coronary artery bypass graft
HCRW: Health and Care Research Wales
HR: Hazard ratio
HRA: Health Research Authority
MLE: Maximum likelihood estimate
NHS: National Health Service
NIHR: National Institute for Health and Research
PATS: Patient Analysis and Tracking System
UK: United Kingdom
VRT: Volume replacement therapy
