# Supplementary material for: Integrating expert opinions with clinical trial data to analyse low-powered subgroup analyses: a Bayesian analysis of the VeRDiCT trial
Source: BMC Med Res Methodol. 2020 Dec 10;20:300. doi: 10.1186/s12874-020-01178-6 (PMC7727208; doi:10.1186/s12874-020-01178-6)
Supplement: Supplementary file 1 — Additional file 1. Elicitation questionnaire for the VeRDiCT trial. [file 12874_2020_1178_MOESM1_ESM.docx]

**Additional file 1** Elicitation questionnaire for the VeRDiCT trial.

**The Trial**

**Preoperative Volume Replacement vs. usual care in Diabetic patients having CABG surgery: a randomised controlled Trial**

**QUESTIONNAIRE**

What is the purpose of the study?

The VeRDICT study was designed to establish the efficacy of preoperative Volume Replacement Therapy (VRT, also called fluid replacement) in diabetic patients undergoing CABG surgery. The trial investigates if VRT reduces renal failure and related complications compared to usual care (i.e. No VRT).

As a clinician involved in the treatment and care of these patients, you have been referred by one of your colleagues to share your opinion about VRT. I would like to ask your opinion on the potential effects of VRT compared to usual care without VRT. I am going to ask you to give your opinion about **the number of patients who** **are** **fit-for-discharge within 6 days after CABG if ever you were treating 100 diabetic patients treated with VRT**. Naturally you may think a wide range of values are plausible. However, you may be confident there may be some areas of the range which are more plausible than others. We have provided an example below on how to provide your opinion.

***X***

**Example:**

For instance, you may think that, out of 100 diabetic patients undergoing CABG surgery, between 15 and 45 could have a normal recovery by being fit-for-discharge within 6 days. You should therefore put a cross under 15 patients to represent the smallest likely value (circled in green below), and a cross under 45 patients to represent the largest likely value in your opinion (circled in blue).

Moreover, you think it is most likely that around 32 patients will have a normal recovery. You should represent the most likely value by putting a cross between 30 and 35 patients (circled in red).

| Example table | Number of patients on VRT fit-for-discharge within 6 days | | | | | | | | | |
| --- | --- | --- | --- | --- | --- | --- | --- | --- | --- | --- |
| Normal recovery patients | <10 | 10 | 15 | 20 | 25 | 30 | 35 | 40 | 45 | >45 |
| **🖝 *Expert opinion*** |  |  | ***X*** |  |  |  |  |  | ***X*** |  |

For each row indicated with the symbol ‘🖝’, you are asked to express this type of opinion by providing the (1) smallest and (2) largest likely values, and (3) the most likely value of the number of patients having a normal recovery (6 days or less in the hospital before being fit-for-discharge).

Thank you very much for taking some time to participate. It should take approximately 10 minutes to answer the 6 questions. Your results will be anonymised. The combination of your thoughts and of other experts will greatly help investigate the effect of VRT in patient subgroups.

You can find out more about the VeRDICT trial or the subgroup analysis by contacting [russell.thirard@bristol.ac.uk](mailto:russell.thirard@bristol.ac.uk)

**Questionnaire**

We are particularly interested in the effect of Volume Replacement Therapy (also referred in practice as fluid replacement) for specific subgroups of diabetic patients:

**(A)** patients at **low (subgroup A1) vs. high risk of renal failure (subgroup A2):** with high risk of renal failure defined by evidence of diabetic nephropathy, microalbuminuria or both.

**(B)** patients being routinely treated with **oral anti-diabetic medication only (subgroup B1) vs. patients given insulin or insulin plus oral anti-diabetic medication (subgroup B2)**.

The expected number of diabetic patients having a normal recovery when treated with usual care (i.e. no VRT) is shown by a vertical red line for each subgroup. This is to help you express your opinion about the effect of VRT. **Please provide (1) the smallest and (2) largest likely values, and (3) the most likely value of the number of patients having a normal recovery by following the example on the first page**. Both mixed opinions (crosses above and below the red line value) and one-sided opinions (either only above or only below the red line value) are absolutely reasonable answers.

***Part (A):* Low (subgroup A1) vs. high risk of renal failure patients (subgroup A2)**

***Q1-*** Suppose 60 of 100 diabetic patients with low risk of post-operative renal failure (subgroup A1) have a normal recovery when receiving usual care (vertical red line). In your opinion, how many of these patients would have a normal recovery if they were treated with VRT?

| Table Q1 |  | | | | | | | | | |  |
| --- | --- | --- | --- | --- | --- | --- | --- | --- | --- | --- | --- |
|  | VRT worse than usual care | | | | | VRT better than usual care | | | | |  |
| Normal recovery patients |  |  |  | <40 40 45 50 55 60 65 70 75 80 >80 |  |  |  |  |  |  |  |
| **🖝 Your opinion** | |  |  |  |  |  |  |  |  |  |  |

***Q2-*** Suppose 35 of 100 diabetic patients with high risk of post-operative renal failure (subgroup A2) have a normal recovery when receiving usual care. In your opinion, how many of these patients would have a normal recovery if they were treated with VRT?

| Table Q2 |  | | | | | | | | | |  |
| --- | --- | --- | --- | --- | --- | --- | --- | --- | --- | --- | --- |
|  | VRT worse than usual care | | | | | VRT better than usual care | | | | |  |
| Normal recovery patients | <15 | 15-20 | 20-25 | 25-30 | 30-35  <15 15 20 25 30 35 40 45 50 55 >55 | 35-40 | 40-45 | 45-50 | 50-55 | >55 |  |
| **🖝 Your opinion** | |  |  |  |  |  |  |  |  |  |  |

***Q3-* Scenario 1.** Suppose that in truth VRT has the same effect as usual care in patients with low risk of renal failure (i.e. 60 patients of subgroup A1 have a normal recovery for both types of care). Given this effect in subgroup A1, what would be your opinion about the effect of VRT compared to usual care for high renal risk patients (subgroup A2)?

**Scenario 2.** Suppose now that in truth VRT increases the number of diabetic patients of subgroup A1 having a normal recovery to 70 patients compared to 60 patients when treated with usual care. Given this effect in subgroup A1, what would be your opinion about the effect of VRT compared to usual care for high renal risk patients (subgroup A2)?

| Table Q3 | |  | | | | | | | | | |  | |
| --- | --- | --- | --- | --- | --- | --- | --- | --- | --- | --- | --- | --- | --- |
|  |  |  | | | | | | | | | |  |  |
|  |  | VRT worse than usual care | | | | | VRT better than usual care | | | | | |  |
| Normal recovery patients | | <15 | 15-20 | 20-25 | 25-30 | 30-35 | 35-40 | 40-45  <15 15 20 25 30 35 40 45 50 55 >55 | 45-50 | 50-55 | >55 | Total | |
| **Your opinion** | |  |  |  |  |  |  |  |  |  |  |  | |
| **🖝** | **Scenario 1** |  |  |  |  |  |  |  |  |  |  |  | |
| **🖝** | **Scenario 2** |  |  |  |  |  |  |  |  |  |  |  | |

**Part (B): Only oral anti-diabetic medication only patients (subgroup B1) vs. patients given insulin or insulin plus oral anti-diabetic medication for routine diabetes management (subgroup B2)**

***Q4-*** Suppose 50 of 100 diabetic patients treated with oral anti-diabetic medication only to manage glycemia prior to surgery (subgroup B1) have a normal recovery when receiving usual care. How many of these 100 patients would have a normal recovery if they were treated with VRT?

| Table Q4 |  | | | | | | | | | |  |
| --- | --- | --- | --- | --- | --- | --- | --- | --- | --- | --- | --- |
|  | VRT worse than usual care | | | | | VRT better than usual care | | | | |  |
| Normal recovery patients | <30 | 30-35 | 35-40 | 40-45  <30 30 35 40 45 50 55 60 65 70 >70 | 45-50 | 50-55 | 55-60 | 60-65 | 65-70 | >70 |  |
| **🖝 Your opinion** |  |  |  |  |  |  |  |  |  |  |  |

***Q5-*** Suppose 40 of 100 diabetic patients treated with insulin or insulin plus oral diabetic medication to manage glycemia prior to surgery (subgroup B2) have a normal recovery when receiving usual care. How many of these 100 patients would have a normal recovery if they were treated with VRT?

| Table Q5 |  | | | | | | | | | |  |
| --- | --- | --- | --- | --- | --- | --- | --- | --- | --- | --- | --- |
|  | VRT worse than usual care | | | | | VRT better than usual care | | | | |  |
| Normal recovery patients | <20 | 20-25 | 25-30 | 30-35 | 35-40 | 40-45 | 45-50 | 50-55 | 55-60 | >60  <20 20 25 30 35 40 45 50 55 60 >60 |  |
| **🖝 Your opinion** |  |  |  |  |  |  |  |  |  |  |  |

***Q6-***
**Scenario 1.** Suppose that in truth, VRT has the same effect as usual care for patients treated with oral anti-diabetic medication only (i.e. 50 patients of subgroup B1 have a normal recovery for both types of care). Given this effect in subgroup B1, what would be your opinion about the effect of VRT compared to usual care for patients treated with insulin only or insulin plus oral medication (subgroup B2)?

**Scenario 2.** Suppose now that in truth, VRT increases the number of diabetic patients having a normal recovery in subgroup B1 to 65 compared to 50 patients when treated with usual care. Given this effect in subgroup B1, what would be your opinion about the effect of VRT compared to usual care for patients treated with insulin only or insulin plus oral medication (subgroup B2)?

| Table Q6 | |  | | | | | | | | | | |
| --- | --- | --- | --- | --- | --- | --- | --- | --- | --- | --- | --- | --- |
|  |  | VRT worse than usual care | | | | | VRT better than usual care | | | | | |
| Normal recovery patients | |  |  | <20 20 25 30 35 40 45 50 55 60 >60 |  |  |  |  |  |  |  |  |
| **Your opinion** | |  |  |  |  |  |  |  |  |  |  |  |
| **🖝** | **Scenario 1** |  |  |  |  |  |  |  |  |  |  |  |
| **🖝** | **Scenario 2** |  |  |  |  |  |  |  |  |  |  |  |

PLEASE ENSURE THAT YOU HAVE PUT 3 CROSSES FOR EACH ROW

**END OF QUESTIONNAIRE**

**Thank you very much**
