# Supplementary figures and images for: Integrating expert opinions with clinical trial data to analyse low-powered subgroup analyses: a Bayesian analysis of the VeRDiCT trial
Source: BMC Med Res Methodol. 2020 Dec 10;20:300. doi: 10.1186/s12874-020-01178-6 (PMC7727208; doi:10.1186/s12874-020-01178-6)

**Additional file 2** R code to run STAN model
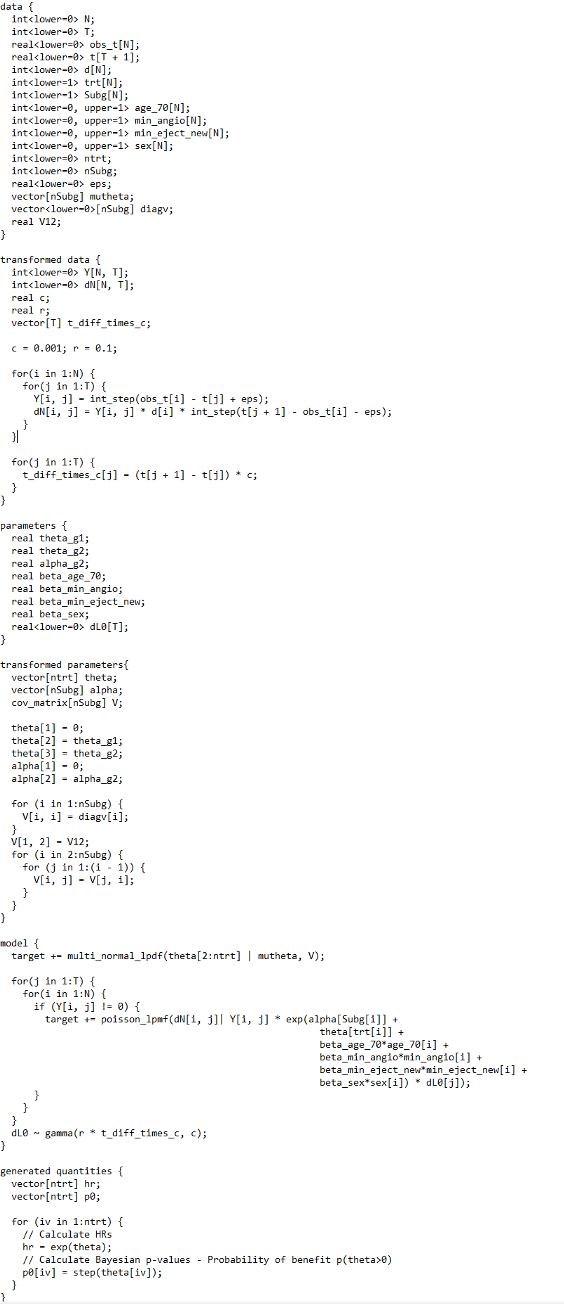

Supplement: Supplementary file 2 — Additional file 2. STAN model code. [file 12874_2020_1178_MOESM2_ESM.docx]
