# Supplementary material for: Integrating expert opinions with clinical trial data to analyse low-powered subgroup analyses: a Bayesian analysis of the VeRDiCT trial
Source: BMC Med Res Methodol. 2020 Dec 10;20:300. doi: 10.1186/s12874-020-01178-6 (PMC7727208; doi:10.1186/s12874-020-01178-6)
Supplement: Supplementary file 3 — Additional file 3. Point estimate and interval results of the Bayesian analysis of the VeRDiCT trial for subgroups A and B. [file 12874_2020_1178_MOESM3_ESM.docx]

**Additional file 3**

Subgroups A: Prior median hazard ratio and 95% interval, maximum likelihood estimate and 95% confidence interval, and posterior median and 95% credible interval using various prior specifications for the risk of renal failure

|  | **Low risk subgroup** | | | **High risk subgroup** | | |
| --- | --- | --- | --- | --- | --- | --- |
|  | **HR** | **95% CI** | **P(benefit)** | **HR** | **95% CI** | **P(benefit)** |
| **Primary analysis** |  |  |  |  |  |  |
| Prior | 1.07 | (0.88, 1.30) | 0.753 | 1.20 | (0.95, 1.51) | 0.938 |
| MLE | 1.14 | (0.63, 2.08) | 0.672 | 0.95 | (0.45, 1.98) | 0.441 |
| Vague prior | 1.15 | (0.62, 2.11) | 0.676 | 0.94 | (0.43, 1.99) | 0.437 |
| Interaction | 1.01 | (0.63, 1.63) | 0.524 | 1.12 | (0.68, 1.83) | 0.673 |
| **Community of priors** |  |  |  |  |  |  |
| Clinical | 1.07 | (0.89, 1.29) | 0.775 | 1.18 | (0.96, 1.45) | 0.940 |
| Sceptical | 1.01 | (0.84, 1.22) | 0.549 | 1.00 | (0.81, 1.24) | 0.506 |
| Interaction-variance | 1.07 | (0.66, 1.73) | 0.616 | 1.06 | (0.64, 1.72) | 0.594 |
| **By expert** |  |  |  |  |  |  |
| A | 0.89 | (0.77, 1.02) | 0.048 | 0.86 | (0.65, 1.14) | 0.151 |
| B | 1.30 | (1.14, 1.48) | 1.000 | 0.98 | (0.78, 1.24) | 0.449 |
| C | 1.00 | (0.88, 1.15) | 0.520 | 1.17 | (1.00, 1.39) | 0.971 |
| D | 1.01 | (0.79, 1.29) | 0.518 | 1.18 | (0.94, 1.47) | 0.923 |
| E | 1.29 | (1.13, 1.48) | 1.000 | 1.56 | (1.32, 1.85) | 1.000 |
| F | 1.22 | (1.14, 1.30) | 1.000 | 1.56 | (1.32, 1.86) | 1.000 |
| G | 0.92 | (0.71, 1.19) | 0.253 | 1.07 | (0.84, 1.37) | 0.720 |
| **Different derivations** |  |  |  |  |  |  |
| Marginal | 1.03 | (0.64, 1.65) | 0.546 | 1.12 | (0.69, 1.84) | 0.689 |
| Conditional mean | 1.05 | (0.66, 1.72) | 0.588 | 1.06 | (0.65, 1.72) | 0.594 |
| *Note:* | | | | | | |
| MLE: maximum likelihood estimate, HR: Hazard ratio, 95% CI: 95 per cent confidence interval for frequentist analyses and 95 per cent credible interval for Bayesian analyses. For the MLE, P(benefit) equals one minus the one-sided P-value in each subgroup for testing the hypothesis of no treatment effect, so that P(benefit)>0.975 is required for 5% significance level. | | | | | | |

Subgroups B: Prior median hazard ratio and 95% interval, maximum likelihood estimate and 95% confidence interval, and posterior median and 95% credible interval using various prior specifications for the type of anti-diabetic medication

|  | **Oral medication subgroup** | | | **Insulin +/- oral medication subgroup** | | |
| --- | --- | --- | --- | --- | --- | --- |
|  | **HR** | **95% CI** | **P(benefit)** | **HR** | **95% CI** | **P(benefit)** |
| **Primary analysis** |  |  |  |  |  |  |
| Prior | 1.15 | (0.99, 1.34) | 0.970 | 1.20 | (0.99, 1.46) | 0.967 |
| MLE | 1.12 | (0.60, 2.07) | 0.638 | 0.96 | (0.46, 2.01) | 0.460 |
| Vague prior | 1.13 | (0.61, 2.08) | 0.647 | 0.96 | (0.45, 2.00) | 0.455 |
| Interaction | 1.04 | (0.64, 1.64) | 0.559 | 1.07 | (0.65, 1.74) | 0.605 |
| **Community of priors** |  |  |  |  |  |  |
| Clinical | 1.15 | (1.00, 1.32) | 0.971 | 1.18 | (0.98, 1.42) | 0.962 |
| Sceptical | 1.01 | (0.88, 1.16) | 0.536 | 1.00 | (0.84, 1.20) | 0.498 |
| **Interaction-variance** | 1.05 | (0.65, 1.71) | 0.583 | 1.04 | (0.64, 1.71) | 0.567 |
| By expert |  |  |  |  |  |  |
| A | 1.01 | (0.88, 1.16) | 0.548 | 0.86 | (0.69, 1.06) | 0.070 |
| B | 1.00 | (0.87, 1.15) | 0.496 | 1.34 | (1.15, 1.56) | 1.000 |
| C | 1.15 | (1.00, 1.32) | 0.977 | 1.16 | (0.99, 1.35) | 0.966 |
| D | 1.15 | (1.00, 1.32) | 0.977 | 1.00 | (0.85, 1.18) | 0.489 |
| E | 1.14 | (1.00, 1.31) | 0.975 | 1.31 | (1.05, 1.64) | 0.988 |
| F | 1.48 | (1.29, 1.69) | 1.000 | 1.51 | (1.28, 1.78) | 1.000 |
| **Different derivations** |  |  |  |  |  |  |
| Marginal | 1.03 | (0.64, 1.64) | 0.547 | 1.07 | (0.65, 1.69) | 0.604 |
| Conditional mean | 1.06 | (0.66, 1.71) | 0.594 | 1.04 | (0.65, 1.69) | 0.561 |
| *Note:* | | | | | | |
| MLE: maximum likelihood estimate, HR: Hazard ratio, 95% CI: 95 per cent confidence interval for frequentist analyses and 95 per cent credible interval for Bayesian analyses. For the MLE, P(benefit) equals one minus the one-sided P-value in each subgroup for testing the hypothesis of no treatment effect, so that P(benefit)>0.975 is required for 5% significance level. | | | | | | |
